# Assessment of Natural Radioactivity Levels and Potential Radiological Risks of Common Building Materials Used in Bangladeshi Dwellings

**DOI:** 10.1371/journal.pone.0140667

**Published:** 2015-10-16

**Authors:** Khandoker Asaduzzaman, Farhana Mannan, Mayeen Uddin Khandaker, Mohideen Salihu Farook, Aeman Elkezza, Yusoff Bin Mohd Amin, Sailesh Sharma, Hasan Bin Abu Kassim

**Affiliations:** 1 Department of Physics, Faculty of Science, University of Malaya, Kuala Lumpur, Malaysia; 2 Institute of Educational Leadership, University of Malaya, Kuala Lumpur, Malaysia; 3 Department of Restorative Dentistry, Faculty of Dentistry, University of Malaya, Kuala Lumpur, Malaysia; 4 Department of Prosthetic Dentistry, Faculty of Dentistry, University of Malaya, Kuala Lumpur, Malaysia; 5 Bangladesh Atomic Energy Commission, Dhaka-1207, Bangladesh; ENEA, ITALY

## Abstract

The concentrations of primordial radionuclides (^226^Ra, ^232^Th and ^40^K) in commonly used building materials (brick, cement and sand), the raw materials of cement and the by-products of coal-fired power plants (fly ash) collected from various manufacturers and suppliers in Bangladesh were determined via gamma-ray spectrometry using an HPGe detector. The results showed that the mean concentrations of ^226^Ra, ^232^Th and ^40^K in all studied samples slightly exceeded the typical world average values of 50 Bq kg^−1^, 50 Bq kg^−1^ and 500 Bq kg^−1^, respectively. The activity concentrations (especially ^226^Ra) of fly-ash-containing cement in this study were found to be higher than those of fly-ash-free cement. To evaluate the potential radiological risk to individuals associated with these building materials, various radiological hazard indicators were calculated. The radium equivalent activity values for all samples were found to be lower than the recommended limit for building materials of 370 Bq kg^-1^, with the exception of the fly ash. For most samples, the values of the alpha index and the radiological hazard (external and internal) indices were found to be within the safe limit of 1. The mean indoor absorbed dose rate was observed to be higher than the population-weighted world average of 84 nGy h^–1^, and the corresponding annual effective dose for most samples fell below the recommended upper dose limit of 1 mSv y^–1^. For all investigated materials, the values of the gamma index were found to be greater than 0.5 but less than 1, indicating that the gamma dose contribution from the studied building materials exceeds the exemption dose criterion of 0.3 mSv y^-1^ but complies with the upper dose principle of 1 mSv y^−1^.

## Introduction

Primordial radionuclides are always present in the environment throughout the world in various isotopic forms, and human beings are constantly exposed to natural sources of ionizing radiation. The natural decay series radionuclides (^238^U, ^232^Th and ^235^U) represent the most significant sources of ionizing radiation on Earth, contributing approximately 83% to the total effective dose received by the global population [1, 2]. The radioactive isotope ^40^K contributes approximately 16% of the annual effective dose experienced by individual members of the global populace due to ionizing radiation [1, 2, 3]. These naturally occurring radionuclides and their associated progenies are the radionuclides that are most commonly found in building materials, and exposure dose result predominantly from these radionuclides [4]. The continuous exposure of human organs to the energetic and particulate forms of radiation released from construction media via the decay chains of ^238^U and ^232^Th in conjunction with ^40^K can cause radiation damage as well as biochemical changes [5].

Similar to other environmental media, building materials with high activity concentrations may increase both indoor and outdoor radiation exposure as well as the internal and external exposure of inhabitants [6–8]. Gamma rays emitted from the uranium and thorium decay series and from ^40^K are the primary sources of whole-body external radiation exposure in buildings, whereas internal radiation exposure, with potential effects on the respiratory area, typically results from inhaling radon and its progeny, which emit alpha particles that are exhaled from building materials into the indoor atmosphere [4, 8–12]. The construction materials used in a home can result in long-term whole-body exposure of the occupants to natural radiation related to ^226^Ra and ^232^Th and their decay products as well as ^40^K, as most people spending approximately 80% of their lifetimes surrounded by building materials at home and/or at the office [7, 8, 10, 13]. The radiological risk to inhabitants may be significant if the materials used for building construction contain elevated levels of radioactivity.

Recently, fly ash (FA), a by-product of coal-fired thermal electric power plants, has become an issue of considerable global interest because of its various applications in the production process for building materials and its substantial economic and environmental value [14]. FA is a technologically important material and is used in the manufacture of building materials and products such as cement, bricks, sheets, concrete, etc. [14, 15]. FA is used in construction work because it increases the strength of concrete, improves sulfate resistance, decreases permeability, reduces the necessary water ratio, and improves the pumpability and workability of the concrete [16]. Like other environmental materials, coal contains natural radionuclides of potassium, thorium and uranium in such trace amounts that they do not create a severe problem in the environment. However, during coal combustion, the majority of the uranium and thorium and their progenies are liberated from the original coal matrix and tend to become enriched in the ashes [15, 17]. After burn-up, most of the radioactive elements are concentrated in the FA, at up to 10 times their original levels [15, 17]. In Bangladesh, 3–4% FA is used for cement production. The radioactivity of building materials and indoor dose rates may be enhanced when industrial by-products such as FA are used in the production process [2, 18–20]. Thus, the characterization and quantification of the natural radioactivity contents of FA and building materials that contain FA are essential for the subsequent estimation of the associated environmental and health hazards.

Radon and its progeny, which emit alpha particles, are the most significant radionuclides that diffuse from building materials into the indoor environments. The radionuclide ^226^Ra, with a half-life of 1600 years, is a source of the radioactive inert gas radon (^222^Rn), which emits alpha and beta particles, followed by gamma radiation. Thus, the concentration of ^226^Ra determines the number of ^222^Rn atoms present in any construction material. Long-term exposure to elevated levels of radon gas and its daughters can lead to functional changes in respiratory organs and may cause lung cancer [6, 21, 22].

As a result of the adverse health effects caused by environmental radiation originating from building materials and the growing social concern regarding this issue, a remarkable number of investigative groups are engaged in the measurement of NORMs (naturally occurring radioactive materials) in such materials at both the national and global levels [4–12, 20–36]. Moreover, various international organizations are engaged in efforts regarding this matter [2, 3, 37–43].

In the present work, radiation characterization was performed for a range of commonly used building materials collected from various suppliers in and around Dhaka city to assess the possible radiological risks to human health due to the use of such materials for building construction. The resulting data serve as a beneficial addition to the pool of already established databases. Such studies can be used to promote the establishment of national standards for the use and management of building materials in light of global recommendations. In addition, the results regarding the measured activity concentrations were compared with the findings from other local studies and from other countries of the world.

## Materials and Methods

No specific permissions were required for this study (locations/activities) because the studied building materials were collected from various manufacturers and suppliers and we do not mention the manufacturers’ names in the manuscript.

### Selection and sampling of building materials

Dhaka (latitude 3°20′N, longitude 101°30′E), the capital of Bangladesh, was selected as the sampling location. Dhaka is one of the most densely populated cities in the world, and the construction industry functions accordingly. The seven most popular brands of locally produced Portland cement were chosen, and at least three samples of each brand were collected from the corresponding dealers. Surface clay brick samples were collected from seven coal-fired brick fields located around the city of Dhaka. Three brick block samples were acquired from each brick field. Raw materials for cement (clinker and gypsum) and the by-products of coal-fired power plants, FA (used as an additive in cement), were imported from abroad and were collected from a cement factory. Sands (red and white) were sampled from housing and other building construction sites and from suppliers in and around Dhaka. Approximately 2–3 kg of each sample from every category of building materials was sampled and transported to the laboratory sample processing room for subsequent investigation. A total of 67 samples of various types of building construction materials were collected.

### Sample pretreatment and analysis for radioactivity measurements

The collected building materials were crushed into small pieces when necessary and dried in an oven at 110°C for 24 hours to remove the moisture from the samples. The items were then ground into fine powder and homogenized by filtering them through a 1-mm sieve. The cement and FA samples were also dried in a hot air oven at 60°C and were sieved to homogenize them. Approximately 400–500 g of each sample was sealed into a Marinelli beaker and stored for a period of 4–5 weeks (more than 7 times the half-lives of ^222^Rn and ^224^Ra) at room temperature to allow secular equilibrium between ^226^Ra and its progeny species to be achieved prior to gamma spectroscopy [44].

The activity concentrations of ^226^Ra, ^232^Th and ^40^K in the samples were determined using a p-type coaxial HPGe γ-ray spectrometer (ORTEC) with a relative efficiency of 28.2% and an energy resolution of 1.67 keV FWHM at the 1332.5 keV peak of ^60^Co shielded by a lead cylinder. The detector linearity was verified using a ^152^Eu gamma-ray-emitting reference source. Energy calibration of the detector was performed using a standard multi-nuclide gamma reference source obtained from the IAEA, and an efficiency calibration was also obtained. The efficiency curves were corrected for the attenuation and self-absorption effects of the emitted gamma photons. For the activity measurements, the samples were counted for a sufficiently long time (86,000 s), and the background counts for the same counting time were subtracted to obtain the net count. The activity concentrations of the radionuclides were obtained using Eq (1), which has been reported elsewhere [5, 45]:

$$A=\frac{N\times 1000}{\varepsilon_{\gamma }\times \rho_{\gamma }\times T_{s}\times M_{s}}$$ (1)

where A is the specific activity in Bq kg^–1^, *N* is the net number of counts in the resulting photo-peak, ε_γ_ is the efficiency of the HPGe detector at the corresponding gamma-ray energy, ρ_γ_ is the intensity at the corresponding gamma-ray energy, T_s_ is the sample counting time in seconds and M_s_ is the weight of the sample in grams.

The gamma-ray spectra of the sample were analyzed to identify and characterize the photopeaks of the ^226^Ra and ^232^Th decay series and that of ^40^K. Because ^226^Ra and ^232^Th are not direct gamma emitters, their activity concentrations were measured via the gamma rays of their progenies. The content of ^226^Ra was measured using the characteristic γ lines of its decay products, including those of ^214^Pb at an energy of 351.92 keV (35.6%), ^214^Bi at 609.32 keV (45.49%), and ^214^Bi at 1764 keV (15.3%). Similarly, the gamma-ray lines at 238.63 keV (46.6%) from ^212^Pb, 583.19 keV (85.0%) from ^208^Tl, 911.16 keV (25.8%) from ^228^Ac, 968.97 keV (16.23%) from ^228^Ac and 2614 keV (35.60%) from ^208^Tl were used to determine the activity concentrations of ^232^Th, whereas ^40^K was measured directly from its own single gamma line at 1460.822 keV (10.66%). The weighted means of the various daughter products were used to obtain the final activity concentrations of ^226^Ra and ^232^Th to reduce the uncertainty of the derived values [6, 44, 46]. The minimum detectable activity concentration (MDAC) of the gamma-ray measurement system was calculated using Eq (2) [5, 9]:

$$MDAC=\frac{K_{\alpha }\times \sqrt{B}}{\varepsilon_{\gamma }\times \rho_{\gamma }\times T_{s}\times M_{s}}$$ (2)

where the statistical coverage factor K_α_ is equal to 1.64 (at the 95% confidence level), B is the number of background counts in the region of interest for a certain radionuclide, ε_γ_ is the efficiency of the HPGe detector at the corresponding gamma-ray energy, ρ_γ_ is the gamma-ray emission probability, T_s_ is the counting time and M_s_ is the dry weight of the sample (kg). The MDACs for the radionuclides of interest were calculated to be 0.35 Bq kg^–1^ for ^226^Ra, 0.64 Bq kg^–1^ for ^232^Th and 2.2 Bq kg^–1^ for ^40^K. The combined uncertainty of the activity concentration was estimated using Eq (3) [8]:

$$\Delta A=A\times \sqrt{{\left(\frac{\Delta N}{N}\right)}^{2}+{\left(\frac{\Delta \varepsilon_{\gamma }}{\varepsilon_{\gamma }}\right)}^{2}+{\left(\frac{\Delta \rho_{\gamma }}{\rho_{\gamma }}\right)}^{2}+{\left(\frac{\Delta m_{s}}{M_{s}}\right)}^{2}+{\left(\frac{\Delta T_{s}}{T_{s}}\right)}^{2}}$$ (3)

where ΔA is the uncertainty of the sample measurement and ΔN, Δε_γ_, Δρ_γ_, Δm_s_ and ΔT_s_ are the uncertainties of the count rate, efficiency, gamma-ray emission probability, sample weight and counting time, respectively.

### Estimation of radiation hazard indicators

To assess the excess gamma radiation originating from building materials, several hazard indices have been suggested by a number of investigators; these measures include the absorbed gamma dose rate in the indoor environment and the corresponding annual effective dose, the radium equivalent activity, the external and internal hazard indices, the alpha index (internal index) and the gamma activity concentration (gamma index) [4–6, 9, 10, 14, 15, 22, 32, 47]. In the present study, the aforementioned hazard indicators were estimated for individuals living in domestic dwellings and for individuals at the workplace to evaluate the potential radiation risks arising from the use of the studied building materials.

### Assessment of the radium equivalent activity (Ra_eq_)

In reality, the relative concentrations of ^226^Ra, ^232^Th and ^40^K are not uniform in environmental media. Accordingly, the distributions of the ^226^Ra, ^232^Th and ^40^K radionuclides were not found to be uniform in the studied building materials. A non-uniform distribution of radioactivity in materials containing Ra, Th and K can be modeled using a general index *Ra*_eq_ (radium equivalent activity) that represents both the total activity of and the radiological risk caused by the building materials [4, 6, 10]. In the present study, Ra_eq_ was computed using Eq (4), which has also been applied by other researchers [4–6, 10, 14, 18, 21, 26, 27]:

$$\begin{array}{c} Ra_{eq}=370\left(\frac{A_{Ra}}{370}+\frac{A_{Th}}{259}+\frac{A_{K}}{4810}\right)\\ \Rightarrow Ra_{eq}=A_{Ra}+1.43A_{Th}+0.077A_{K} \end{array}$$ (4)

where *A*_Ra_, *A*_Th_ and *A*_K_ (in Bq kg^–1^) are the activity concentrations of ^226^Ra, ^232^Th and ^40^K, respectively. Eq (4) is based on the estimation that 370 Bq kg^–1^ of ^226^Ra, 259 Bq kg^–1^ of ^232^Th and 4810 Bq kg^–1^ of ^40^K each produce an identical γ-ray dose rate [4–6, 10, 14, 18].

### Absorbed dose rate and annual effective dose

The external absorbed dose rate *D* (nGy h^–1^) delivered by the radionuclides under investigation to the general public in the outdoor air was calculated using Eq (5), which has been presented by a number of researchers [4, 5, 27, 28]:

$$D_{out}=0.427\times A_{Ra}+0.662\times A_{Th}+0.0432\times A_{K}$$ (5)

where A_Ra_, A_Th_ and A_K_ are the activity concentrations of ^226^Ra, ^232^Th and ^40^K, respectively, in Bq kg^–1^. Indoor exposure to gamma rays is naturally higher than outdoor exposure because predominantly earth-originating materials are used in building construction. When the duration of occupancy is taken into account, indoor exposure becomes more significant. Because the investigated materials (e.g., brick, cement and sand) are extensively used as construction materials in homes, it is important to evaluate their effects on indoor exposure. Considering that the indoor dose contribution is 1.4 times higher than the outdoor dose contribution, the gamma dose D_in_ (nGy h^–1^) in the indoor environment that is delivered by radionuclides (gamma discharge from ^226^Ra, ^232^Th and ^40^K) in the investigated structural construction materials was assessed using Eq (6) [40, 48]:

$$D_{in}=1.4\times D_{out}$$ (6)

The corresponding annual effective dose, E_in_ (mSv y^–1^), was evaluated using a value of 0.7 SvGy^–1^ [40] for the conversion factor from the absorbed dose in air to the effective dose received by an adult and a value of 0.8 for the indoor occupancy factor to represent the fact that worldwide, people spend an average of approximately 80% of their time indoors [6–10, 13]. Thus, the annual effective dose (mSv y^–1^) received by a building occupant due to the activity in the building materials was estimated using Eq (7) [4, 6, 8–10, 21]:

$$E_{in}=D_{in}(nGyh^{-1})\times 8760h\times 0.7Svy^{-1}\times 0.8\times 10^{-6}$$ (7)

### Gamma activity concentration index or gamma index (Iγ)

To limit the excess gamma radiation originating from building materials, an index, i.e., the gamma index (external index), is defined for use as a screening tool for categorizing materials used in construction [6, 9, 10]. For a typical building material, this gamma index can be estimated using Eq (8), as recommended by the European Commission [37]:

$$I_{\gamma }=\frac{A_{Ra}}{300Bqkg^{-1}}+\frac{A_{Th}}{200Bqkg^{-1}}+\frac{A_{K}}{3000Bqkg^{-1}}$$ (8)

where A_Ra_, A_Th_ and A_K_ are the measured activity concentrations in Bq kg^-1^ for ^226^Ra, ^232^Th and ^40^K, respectively; it is assumed that activity concentrations of 300 Bq kg^–1^ for ^226^Ra, 200 Bq kg^–1^ for ^232^Th and 3000 Bq kg^–1^ for ^40^K each produce the same gamma dose rate. For a structural material, the exemption dose criterion (annual effective dose) of 0.3 mSv y^-1^ corresponds to a gamma index of Iγ ≤ 0.5, whereas the upper dose criterion of 1 mSv y^-1^ is satisfied for Iγ ≤ 1 [9, 37].

### Alpha index (internal index, Iα)

Excess alpha radiation caused by the inhalation of radon liberated from building materials can be estimated using the alpha index (Iα), which has been applied by various researchers [5, 6, 9, 47]:

$$I_{\alpha }=\frac{A_{Ra}}{200Bqkg^{-1}}$$ (9)

where A_Ra_ is the activity concentration of the alpha emitter ^226^Ra (Bq kg^‒1^). Radon exhalation from a given construction material may lead to indoor radon concentrations that exceed the recommended action level of 200 Bq m^−3^ if the activity concentration of ^226^Ra in the material exceeds a value of 200 Bq kg^‒1^ [6, 9, 42, 47]; thus, the safe limit is defined by an alpha index of less than or equal to unity.

### External (H_ex_) and internal (H_in_) hazard indices

The intent of applying these two health hazard indices, which are useful herein for the characterization of building materials, is to set a limiting value on the acceptable equivalent dose [41] as recommended in a report by the ICRP (1990) [10, 21]. To limit the radiation dose from a construction material to 1.5 mSv y^−1^, the value of H_ex_ must be less than unity [5, 7, 10, 21, 27, 28]. In the present study, H_ex_ was calculated using Eq (10), as formulated by Beretka and Mathew (1985) [29]:

$$H_{ex}=\frac{A_{Ra}}{370}+\frac{A_{Th}}{259}+\frac{A_{K}}{4810}$$ (10)

where *A*_Ra_, *A*_Th_ and *A*_K_ represent the measured activity concentrations in Bq kg^−1^ for ^226^Ra, ^232^Th and ^40^K, respectively.

Inhaled radon and its short-lived progeny also represent a risk to the respiratory organs. Internal exposure to radon and its progeny can be quantified using the index *H*_in_, which is estimated using Eq (11) [5, 27, 29]:

$$H_{in}=\frac{A_{Ra}}{185}+\frac{A_{Th}}{259}+\frac{A_{K}}{4810}$$ (11)

For the utilization of a building material to be considered safe, *H*_in_ must be less than 1 [5, 10, 21].

### Significance of various hazard indices

^238^U and ^232^Th decay series radionuclides and also the ^40^K are common elements to all earth born materials. All radioactive progenies of ^238^U and ^232^Th parents emit α or β particles followed by γ-rays until their end-up to stable ^208^Pb and ^206^Pb. However, majority of the emitted α and β particles cannot come out from the sample matrix to the outside environment due to their low penetration power. On the other hand, most of the γ-rays can easily penetrate the sample matrix and enter into the building atmosphere.

Since γ-rays emitted from building material can easily travel long distances within the surrounding environment, human beings may continuously exposed by gamma radiation and adverse health effects may occurred via extended period of exposure. Thus, the representative gamma-index, absorbed dose rate and annual effective dose find great significance to understand the health hazards from gamma-radiation exposures. Furthermore, external hazard index (H_ex_) is often used to characterize the building materials via set up a limiting value on the acceptable equivalent dose (or to limit the external γ-radiation dose),

Generally, the distribution of ^226^Ra, ^232^Th and ^40^K in environmental sample including construction materials are not uniform. In order to overcome the non-uniformity of the radionuclides, a common index called “radium equivalent activity (Ra_eq_)” is used to obtain the representing activity and also to assess the radiological hazard caused by the building materials.

Moreover, some of our investigated materials such as fly-ash and cement can easily be inhaled by people and then the α and β emitters (sub-series headed by ^226^Ra and ^228^Ra) can easily be attached to the living cell of the respiratory organs, causes the cell damage as well as create cancer. For these seasons internal hazard index (H_in_) and alpha index (I_α_) are often used to characterized building materials [5, 6, 9, 10, 21, 27, 29, 47].

## Results and Discussion

The results obtained for the activity concentrations of ^226^Ra, ^232^Th and ^40^K in the various building material samples are presented in Table 1. The mean concentrations in the analyzed building material samples ranged from 49.4±3.0 to 60.5±2.1 Bq kg^‒1^ for ^226^Ra, from 64.7±2.6 to 82.0±3.6 Bq kg^‒1^ for ^232^Th and from 927.2±13.8 to 1080.3±12.7 Bq kg^‒1^ for ^40^K. The highest mean values of radionuclide concentration were found as 60.5±2.1 Bq kg^‒1^ in the cement samples for ^226^Ra, 82.0±3.6 Bq kg^‒1^ in the red sand for ^232^Th and 1080.3±12.7 Bq kg^‒1^ in the brick samples for ^40^K, whereas the lowest mean values of ^226^Ra, ^228^Ra, and ^40^K concentrations were observed in white sand (49.4±3.0 Bq kg^‒1^), cement (64.7±2.6 Bq kg^‒1^) and white sand (927.2±13.8 Bq kg^‒1^), respectively. The mean activity levels of ^226^Ra in cement, brick and sand samples were found to be within the typical global range (17–60 Bq kg^‒1^) [5]. By contrast, the mean activity concentrations of ^232^Th and ^40^K in the same building material samples were significantly higher than the typical global ranges (11–64 Bq kg^‒1^ for ^232^Th and 140–850 Bq kg^‒1^ for ^40^K) [5]. The variations in activity concentration among the building materials may be attributed to their radioactive mineral content and the geological, geochemical and geographical origins of the raw materials, among other factors [21]. The activity concentrations of thorium were observed to be greater than those of uranium/radium, consistent with the fact that the abundance of thorium is approximately 1.5 times higher than that of uranium in the earth’s crust [49]. Moreover, Molla (1980) has reported that the thorium level throughout Bangladeshi soil is, in general, higher than that of uranium [50]. For all types of building material samples, with the exception of the white sand, the mean values of the ^226^Ra, ^232^Th and ^40^K concentrations somewhat exceeded the corresponding typical world values of 50 Bq kg^−1^, 50 Bq kg^−1^ and 500 Bq kg^−1^, respectively, as compiled in the UNSCEAR-1993 report [47, 51].

**Table 1:** Radioactivity concentrations of ^226^Ra, ^232^Th and ^40^K radionuclides in the various building media under study.

Sample	Radioactivity concentration (Bq kg^–1^)		
	^226^Ra	^232^Th	^40^K
**Cement**			
CMB-1	82.8±2.2	63.5±2.2	946.5±12.4
CMB-2	74.0±2.0	75.4±2.4	927.2±12.2
CMB-3	41.0±2.4	58.8±4.6	915.4±12.0
CMB-4	64.5±2.0	64.4±2.2	945.4±12.3
CMB-5	55.4±1.9	58.9±2.3	955.3±12.1
CMB-6	52.2±2.0	67.8±2.4	941.9±12.2
CMB-7	53.6±1.9	64.2±2.4	1033.3±12.7
**AM±SD**	**60.5±2.1**	**64.7±2.6**	**952.2±12.6**
**Fly ash**			
FA-1	118.7±6.9	181.0±8.2	1557.0±29.7
FA-2	126.1±8.4	178.5±12.1	1630.3±30.0
FA-3	121.3±4.5	138.6±6.5	1367.4±14.8
FA-4	109.5±4.7	167.7±7.7	1523.5±21.5
FA-5	113.3±6.3	120.7±8.3	1238.4±17.8
**AM±SD**	**117.8±6.2**	**157.3±8.6**	**1463.3±22.8**
**Clinker**			
CKR-1	50.4±4.2	68.8±5.7	833.7±15.7
CKR-2	52.7±3.6	76.7±3.9	871.8±16.3
CKR-3	46.4±5.3	81.5±4.6	863.7±18.5
**AM±SD**	**49.8±4.4**	**75.7±4.7**	**856.4±16.8**
**Gypsum**			
GPM-1	53.9±4.5	90.2±4.5	1113.9±20.8
GPM-2	63.8±3.5	93.9±4.8	1101.6±20.5
GPM-3	57.4±3.7	89.6±5.5	1087.8±17.6
**AM±SD**	**58.4±3.7**	**91.2±3.8**	**1101.1±19.6**
**Brick**			
BRB-1	59.6±2.0	77.9±2.3	1064.4±12.9
BRB-2	58.5±2.3	75.6±2.8	1102.6±12.5
BRB-3	41.4±1.5	64.9±3.5	986.5±13.7
BRB-4	47.6±3.4	68.2±2.6	1126.2±13.7
BRB-5	67.6±2.8	84.8±3.5	1082.7±11.4
BRB-6	53.8±1.8	71.6±3.7	1043.1±11.5
BRB-7	74.4±1.9	87.6±2.4	1156.8±12.7
**AM±SD**	**57.5±2.2**	**75.8±2.9**	**1080.3±12.7**
**White sand**			
WSD-1	45.4±2.4	60.8±2.6	832.4±10.3
WSD-2	33.3±1.4	79.3±4.7	925.8±18.4
WSD-3	63.3±4.9	82.5±2.8	1046.1±15.6
WSD-4	56.5±3.1	71.2±4.2	936.2±11.5
WSD-5	48.5±3.1	64.3±3.2	895.4±13.2
**AM±SD**	**49.4±3.0**	**71.6±3.5**	**927.2±13.8**
**Red sand**			
RSD-1	46.0±4.5	88.2±3.9	889.3±18.5
RSD-2	58.5±2.8	65.6±5.2	1086.2±9.5
RSD-3	53.7±4.2	77.3±2.4	1079.6±14.3
RSD-4	70.6±1.2	84.2±3.0	1136.2±12.6
RSD-5	67.4±3.9	94.6±3.6	986.6±14.8
**AM±SD**	**59.2±3.3**	**82.0±3.6**	**1035.6±14.0**

**AM±SD denotes** arithmetic mean±standard deviation.

The mean concentrations of ^226^Ra, ^232^Th and ^40^K in the clinker samples tested in the present study were found to be very similar to those observed in the cement samples because Portland cement is made by milling clinker with the simultaneous addition of only approximately 5% gypsum; therefore, the chemical composition of the clinker ‘‘dictates” the radionuclide content in the cement [40]. Meanwhile, the concentrations of ^226^Ra, ^232^Th and ^40^K determined in the present study for gypsum were, to some extent, higher than those in the studied cement samples.

The activity concentrations of the FA were found to be significantly higher (117.8±6.2 Bq kg^–1^ for ^226^Ra, 157.3±8.5 Bq kg^–1^ for ^232^Th and 1463.3±22.8 Bq kg^–1^ for ^40^K) than the concentrations in the building materials and the ingredients of cement. The obtained results are consistent with those available in the literature for cement and coal FA [40]. The ratio of the ^226^Ra activity in the Portland cement to that in the coal FA in the present study was found to be 0.51; this is slightly higher than the ratios reported in the literature, which typically vary in the range between 0.24 and 0.40 [40, 52]. In the present study, FA was used (4‒7%) in the production of the cement brands represented by samples CMB-1 and CMB-2 (Table 1). It is clear that these two samples contained relatively higher radioactivity levels (especially ^226^Ra) than did the samples of FA-free cement brands. Stoulos et al. (2003) [53] have shown that the use of FA in cement production increases the radioactivity of the cement (Table 2).

**Table 2:** Radiation hazard indicators for ^226^Ra, ^232^Th and ^40^K radionuclides in the various building materials under study.

Sample	Radium equivalent activity (Bq kg^−1^)	Hazard index		Indoor absorbed dose rate (nGy h^−1^)	Annual effective dose (mSv y^−1^)	Alpha index	Gamma index
	Ra_eq_	H_ex_	H_in_	D_in_	E_in_	Iα	Iγ
**Cement**							
CMB-1	246.3	0.67	0.89	165.6	0.81	0.41	0.91
CMB-2	253.0	0.68	0.88	170.2	0.83	0.37	0.93
CMB-3	195.4	0.53	0.64	134.4	0.66	0.21	0.74
CMB-4	229.2	0.62	0.79	155.4	0.76	0.32	0.85
CMB-5	213.1	0.58	0.73	145.5	0.71	0.28	0.80
CMB-6	221.6	0.60	0.74	151.0	0.74	0.26	0.83
CMB-7	224.8	0.61	0.75	154.0	0.76	0.27	0.84
AM±SD	226.2±19.5	0.61±0.05	0.77±0.09	153.7±12	0.75±0.06	0.30±0.07	0.84±0.06
**Fly ash**							
FA-1	486.5	1.3	1.7	332.9	1.63	0.59	1.8
FA-2	495.5	1.4	1.7	339.4	1.66	0.63	1.9
FA-3	415.2	1.1	1.5	283.7	1.39	0.61	1.6
FA-4	456.0	1.3	1.6	313.0	1.54	0.55	1.7
FA-5	372.6	1.0	1.3	254.5	1.25	0.57	1.4
AM±SD	245.2±51.3	1.2±0.16	1.6±0.17	304.7±35.5	1.49±0.17	0.59±0.03	1.7±0.19
**Clinker**							
CKR-1	207.2	0.60	0.70	144.3	0.71	0.25	0.79
CKR-2	223.5	0.60	0.80	155.3	0.76	0.26	0.85
CKR-3	229.4	0.62	0.75	155.5	0.76	0.23	0.85
AM±SD	220.0±11.5	0.61±0.01	0.75±0.05	151.7±6.4	0.74±0.03	0.25±0.02	0.83±0.03
**Gypsum**							
GPM-1	261	0.73	0.87	183.2	0.90	0.27	1.0
GPM-2	275.1	0.76	0.94	191.8	0.94	0.32	1.1
GPM-3	269.3	0.73	0.88	183.1	0.90	0.29	1.0
AM±SD	268.5±7.1	0.74±0.02	0.90±0.03	186.0±5.0	0.91±0.02	0.29±0.03	1.0±0.06
**Brick**							
BRB-1	252.7	0.68	0.84	172.2	0.84	0.30	0.94
BRB-2	151.4	0.68	0.84	171.7	0.84	0.29	0.94
BRB-3	209.9	0.57	0.68	144.6	0.71	0.21	0.79
BRB-4	231.7	0.63	0.75	159.8	0.78	0.24	0.88
BRB-5	271.9	0.73	0.92	184.5	0.91	0.34	1.0
BRB-6	236.3	0.64	0.78	161.6	0.79	0.27	0.89
BRB-7	288.4	0.78	0.98	195.6	0.96	0.37	1.07
AM±SD	248.9±26.1	0.67±0.07	0.83±0.1	170.0±16.8	0.83±0.08	0.29±0.06	0.93±0.09
**Sand**							
WSD-1	196.4	0.53	0.65	133.8	0.66	0.23	0.73
WSD-2	217.8	0.59	0.68	149.4	0.73	0.17	0.82
WSD-3	261.7	0.71	0.88	177.6	0.87	0.32	0.97
WSD-4	230.3	0.62	0.78	156.4	0.77	0.28	0.86
WSD-5	209.3	0.57	0.70	142.7	0.70	0.24	0.78
AM±SD	223.1±25	0.60±0.07	0.74±0.09	152.0±16.6	0.75±0.08	0.25±0.06	0.83±0.09
RSD-1	240.5	0.65	0.77	163.0	0.80	0.23	0.89
RSD-2	235.7	0.64	0.80	161.4	0.79	0.29	0.88
RSD-3	247.2	0.67	0.81	169.0	0.83	0.27	0.93
RSD-4	278.3	0.75	0.94	189.0	0.93	0.35	1.0
RSD-5	278.4	0.75	0.93	187.6	0.92	0.34	1.0
AM±SD	256.0±20.8	0.69±0.05	0.85±0.08	174.0±13.3	0.85±0.07	0.30±0.05	0.95±0.06

AM±SD denotes arithmetic mean±standard deviation.

To keep the external doses below 1.5 mSv y^–1^, the maximum values of radium equivalent activity (*Ra*_eq_) would need to be less than 370 Bq kg^–1^ [6, 7, 10, 28, 38]. In the present study, the *Ra*_eq_ values for all samples (with the exception of the FA) were found to be lower than the safe limit value of 370 Bq kg^–1^ suggested by the OECD (1979) [6, 10, 28, 38].

**Fig 1:**
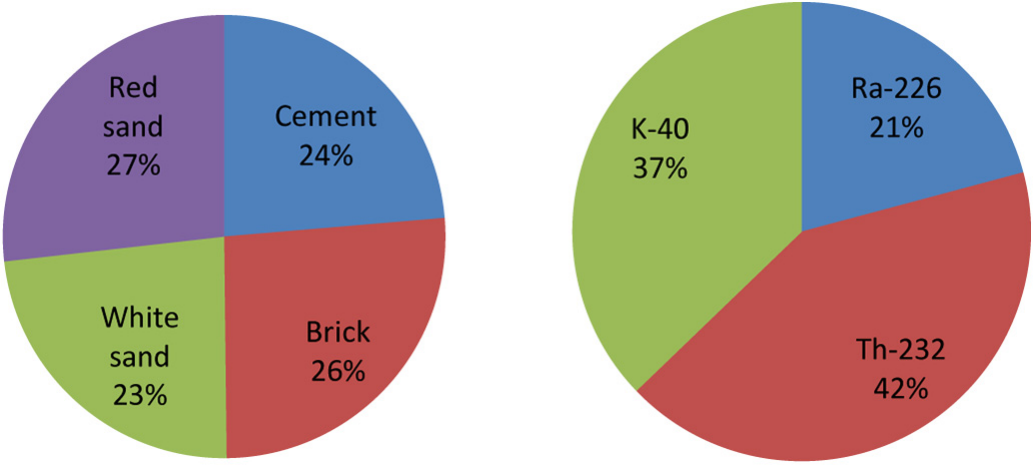
Dose contribution due to the content of ^226^Ra, ^232^Th and ^40^K in building materials to the inhabitants.

The lowest indoor absorbed dose rate (arithmetic mean ± standard deviation) of 152.0±16.6 nGy h^–1^ was obtained for the white sand samples, followed by the cement (153.7±12.0 nGy h^–1^), whereas the maximum value of 174.0±13.3 nGy h^–1^ was observed for the red sand samples, followed by the brick samples (170.0±16.8 nGy h^–1^). The mean indoor absorbed dose levels in the cement, brick and sand samples were found to fall within the typical worldwide range (20–200 nGy h^–1^) [9, 40], although they were approximately two times higher than the quoted population-weighted average value of 84 nGy h^–1^ [9, 40]. Meanwhile, the annual effective dose values in the cement, brick, white sand and red sand samples were found to be 0.75±0.06, 0.83±0.08, 0.75±0.08 and 0.85±0.07 mSv, respectively. These values are well below the maximum allowable dose equivalent limit of 1 mSv y^–1^ recommended by the ICRP (1990) [4, 6, 21, 22, 41]. Fig 1 shows the doses to inhabitants originating from the contents of ^226^Ra, ^232^Th and ^40^K in commonly used building materials. Among the investigated materials, red sand (27%) is the highest contributor to the annual indoor effective dose, followed by brick (26%), cement (24%) and white sand (23%). Among the radionuclides, ^232^Th is the predominant contributor to the dose in the indoor environment, with a contribution of approximately 42% of the total estimated dose, followed by ^40^K (37%) and ^226^Ra (21%).

For a building material, the exemption dose criterion 0.3 mSv y^−1^ corresponds to the gamma index Iγ≤0.5, whereas the dose criterion of 1 mSv y^−1^ is satisfied for Iγ≤1 [9, 37]. According to this dose criterion, materials with Iγ≥1 should be avoided in building construction because these values correspond to dose rates higher than 1 mSv y^−1^ [37]. For all samples under study, it was found that Iγ>0.5 but Iγ≤1, indicating that the gamma dose contributions from the studied building materials exceeded the exemption dose criterion of 0.3 mSv y^−1^ while remaining lower than the upper dose criterion of 1 mSv y^−1^, with the exception of two brick samples, two red sand samples, and all gypsum and FA samples. The evaluated alpha index (Iα) values were well below the recommended upper level of 1 for internal exposure [5, 6, 9, 22, 47]. The ^226^Ra activity concentrations in the investigated samples (Table 1) were significantly below 200 Bq kg^‒1^, indicating that the indoor radon concentrations did not exceed the recommended activity level of 200 Bq m^−3^.

The estimated values of the external and internal hazard indices (Table 2) for all types of building material samples analyzed in this work (except FA) were found to be less than the recommended limit of 1 for the safe utilization of a material in the construction of dwellings [5, 10, 21, 48].

In Table 3, the mean values of the activity concentrations of ^226^Ra, ^232^Th and ^40^K determined in the present study for the cement, brick and sand samples are compared with the corresponding literature values determined in other countries. Overall, the mean activity levels of the examined building material samples were comparable to and greater than those from other countries. The activity levels vary from one country to another, which can be attributed to differences in the contents of radioactive minerals and in the geological, geochemical and geographical origins of the raw materials, among other factors. This fact is important to consider in the section of suitable materials for use in building construction, especially those that exhibit large variations in their activities.

**Table 3:** Comparison of the average activity concentrations of the studied building materials with other published data.

Country	Radioactivity concentration (Bq kg^−1^)			References
	^226^Ra	^232^Th	^40^K	
**Cement**				
Present study	60.5±2.1	64.7±2.6	952.2±12.6	−
Pakistan (Punjab)	37±3	28±3	200±14	Rahman et al., 2013 [4]
India (South-West)	54±13	65±10	440±91	Khandaker et al., 2012 [5]
Turkey (Manisa)	55.64±0.75−86.71±1.64	LLD−7.19±0.10	348.17±10.00−265.75±6.40	Erees et al., 2006 [7]
Turkey (Adana)	49.8±5.8	17.3±2.2	246.2±20.8	Solak et al., 2014 [9]
China (Xian)	68.3±3.6	51.7±5.4	173.8±8.6	Xinwei, 2005 [11]
China (Urumqi)	29.1±2.1	15.8±1.9	333.2±83.2	Ding et al., 2013 [12]
Egypt (Qena)	134±67	88±35	416±162	Ahmed, 2005 [28]
Vietnam	39.86±17.43	25.46±4.69	243.5±62.2	Le et al., 2011 [33]
Greece (FA Add. <3%)	20±5	13±3	247±68	Stoulos et al., 2003 [53]
Greece (FA Add. <20%)	92±33	31±10	310±60	Stoulos et al., 2003 [53]
Bangladesh (Dhaka)	61.1±0.8	79.9±1.2	1132.6±17.3	Roy et al., 2005 [54]
Qatar	23.4±0.6	12.2±0.2	158.8±4.3	Sulaiti et al., 2011 [55]
Nigeria	43.8	21.5	71.7	Ademola, 2008 [56]
Cuba	23±7	11±3	467±85	Flores et al., 2008 [57]
South Korea	34.5±1.7	19.4±1.5	241±6.7	Lee et al., 2001 [58]
India (Tamil-nadu)	37.98	34.87	188.13	Ravisankar et al., 2012 [59]
**Brick**				
Present study	57.5±2.2	75.8±2.9	1080.3±12.7	−
Pakistan (Punjab)	58±4	84±5	542±18	Rahman et al., 2013 [4]
India (South-West)	21±4	21±3	290±20	Khandaker et al., 2012 [5]
Turkey (Manisa)	42.4	16.1	553.3	Erees et al., 2006 [7]
Turkey (Adana)	5.3±15.7	25.8±4.7	404.8±43.7	Solak et al., 2014 [9]
China (Xian)	58.6±4.7	50.4±3.5	713.9±8.2	Xinwei, 2005 [11]
China (Urumqi)	49.3±2.9	44.5±1.7	860.4±65.7	Ding et al., 2013 [12]
Egypt (Qena)	33±20	37±17	511±158	Ahmed, 2005 [28]
Vietnam	64.35±23.78	77.57±32.49	589.0±171.8	Le et al., 2011 33]
Italy	20±2−110±9	25±2−97±8	160±10−680±60	Righi and Bruzzi, 2006 [47]
Greece	35±11	45±15	710±165	Stoulos et al., 2003 [53]
Bangladesh (Dhaka)	43.4±2.7−45.9±2.8	97.1±6.7−105.6±7.2	1550.8±119.2−1564.2±120.1	Roy et al., 2005 [54]
Cuba	57±16	12±10	857±759	Flores et al., 2008 [57]
South Korea	33.3	79.8	698	Lee et al., 2001 [58]
India (Tamil-nadu)	18.3	19.4	238.4	Ravisankar et al., 2012 [59]
**Sand**				
Present study: White	49.4±3.0	71.6±3.5	927.2±13.8	−
Red	59.2±3.3	82.0±3.6	1035.6±14.0	
Pakistan (Punjab)	24±2	39±3	462±16	Rahman et al., 2013 [4]
Turkey (Manisa) (max^m^)	1559.10	142.48	1711.47	Erees et al., 2006 [7]
Turkey (Adana)	38.8±10.0	29.5±11.3	471.4±101.2	Solak et al., 2014 [9]
China (Xian)	40.7±4.3	21.5±5.6	302.6±3.4	Xinwei, 2005 [11]
China (Urumqi)	22.4±1.9	25.1±2.5	789.3±45.0	Ding et al., 2013 [12]
Vietnam	26.74±30.30	39.77±61.70	506.9±317.1	Le et al., 2011 [33]
Greece	18±7	17±10	367±204	Stoulos et al., 2003 [53]
Bangladesh (Dhaka)	51.3±1.4	135.0±4.0	1592.2±54.6	Roy et al., 2005 [54]
Qatar	13.2±0.3	3.34±0.05	225.5±6.1	Sulaiti et al., 2011 [55]
Cuba	17±4	16±6	208±104	Flores et al., 2008 [57]
South Korea	28.98	56.37	1008	Lee et al., 2001 [58]
India (Tamil-nadu)	2.27	21.72	352.8	Ravisankar et al., 2012 [59]

## Conclusions

Materials that are locally produced and extensively used for the construction of buildings by Bangladeshi inhabitants were examined to assess their radioactivity levels. The mean activity concentrations of ^232^Th and ^40^K in the cement, brick and sand samples were considerably higher than the typical worldwide ranges, whereas the levels of ^226^Ra in the same samples were found to be within the typical global range. The results show that the use of fly ash in cement production increases the radioactivity of the cement (especially ^226^Ra). The radium equivalent activities of the various building materials were found range from 223.1±25.0 to 256.0±20.8 Bq kg^‒1^, well below the recommended safe limit of 370 Bq kg^‒1^. The absorbed dose rates in the indoor environment originating from the building materials were found to be higher than the quoted global average value, whereas the corresponding annual effective doses were lower than the upper dose limit of 1 mSv y^–1^ recommended by international organizations. The gamma dose contributions from the studied building materials exceeded the exemption dose criterion of 0.3 mSv y^−1^ but were lower than the upper dose criterion of 1 mSv y^−1^. Moreover, the values of the alpha index and the radiological hazard (external and internal) indices were found to be within the safe limit of 1. The data reported herein can be regarded as base values for the distributions of natural series radionuclides in cement, brick and sand in the studied region and may be used as reference information for environmental radioactivity monitoring with the intent of minimizing population exposure to ensure a safer living environment.
